# SAWRPI: A Stacking Ensemble Framework With Adaptive Weight for Predicting ncRNA-Protein Interactions Using Sequence Information

**DOI:** 10.3389/fgene.2022.839540

**Published:** 2022-02-28

**Authors:** Zhong-Hao Ren, Chang-Qing Yu, Li-Ping Li, Zhu-Hong You, Yong-Jian Guan, Yue-Chao Li, Jie Pan

**Affiliations:** ^1^ School of Information Engineering, Xijing University, Xi’an, China; ^2^ School of Computer Science, Northwestern Polytechnical University, Xi’an, China

**Keywords:** ncRNA-protein interactions, ncRNA, ensemble learning, sequence analysis, natural language processing

## Abstract

Non-coding RNAs (ncRNAs) take essential effects on biological processes, like gene regulation. One critical way of ncRNA executing biological functions is interactions between ncRNA and RNA binding proteins (RBPs). Identifying proteins, involving ncRNA-protein interactions, can well understand the function ncRNA. Many high-throughput experiment have been applied to recognize the interactions. As a consequence of these approaches are time- and labor-consuming, currently, a great number of computational methods have been developed to improve and advance the ncRNA-protein interactions research. However, these methods may be not available to all RNAs and proteins, particularly processing new RNAs and proteins. Additionally, most of them cannot process well with long sequence. In this work, a computational method SAWRPI is proposed to make prediction of ncRNA-protein through sequence information. More specifically, the raw features of protein and ncRNA are firstly extracted through the k-mer sparse matrix with SVD reduction and learning nucleic acid symbols by natural language processing with local fusion strategy, respectively. Then, to classify easily, Hilbert Transformation is exploited to transform raw feature data to the new feature space. Finally, stacking ensemble strategy is adopted to learn high-level abstraction features automatically and generate final prediction results. To confirm the robustness and stability, three different datasets containing two kinds of interactions are utilized. In comparison with state-of-the-art methods and other results classifying or feature extracting strategies, SAWRPI achieved high performance on three datasets, containing two kinds of lncRNA-protein interactions. Upon our finding, SAWRPI is a trustworthy, robust, yet simple and can be used as a beneficial supplement to the task of predicting ncRNA-protein interactions.

## Introduction

Protein is the main carrier of cellular activities. Human proteins are translated from less than 2% of genome, but more than 80% of genome has biochemical functions (Djebali et al., 2012; Pennisi 2012), which accounts for the large number of non-coding RNA (ncRNA), known as the RNA with little or without ability of encoding proteins, have biological functions. There is an emerging recognition of RNA that any transcripts can have intrinsic functions (Han et al., 2019). Long non-coding RNA (lncRNA) is a class of transcribed RNA molecules with no ability of encoding proteins, which has more than 200 nucleotides (Prensner and Chinnaiyan 2011; Volders et al., 2013) and more than 70% of ncRNA are lncRNAs (Yang et al., 2014). Massive amount of lncRNA means largely precious biological information is waiting for mining. It has demonstrated that various complex diseases have strong correlation with lncRNA, like Alzheimer (Ng et al., 2013) and lung cancer (Shi et al., 2015). Moreover, biological studies revealed that lncRNA plays important roles in gene regulation, splicing, translation, chromatin modification and poly-adenylation (Wang and Chang 2011; Nie et al., 2012; Zeng et al., 2017). However, it is still largely unknown that the biological functions of most ncRNAs. And on account of interactions between ncRNA and RNA binding proteins (RBPs) is a critical way of ncRNA executing biological functions (Zhu et al., 2013), to the understanding biological functions of ncRNA, identifying ncRNA-protein interactions is a crucial step. Wet-lab experiments have been designed to verify ncRNA-protein interactions, like RNAcompete (Ray et al., 2009), RIP-Chip (Keene et al., 2006), and HITS-CLIP (Darnell 2010). While, in the post-genomic era, much time is used to hand-tune carefully putatively bound sequences for high-throughput technologies and it is costly to determine complex sequence structure of them (Alipanahi et al., 2015). Additionally, wet experiments have no ability to examine ncRNA-protein interactions efficiently and effectively because of the large number of unexplored interactions. Due to experimental methods are costly, time-consuming and localized, and sequences of RNA and protein carry sufficient information for predicting interaction between them (Ray et al., 2009; Alipanahi et al., 2015), many computational models have been proposed as alternative methods to overcome the drawbacks of ncRNA-protein interactions prediction.

Nowadays, two kinds of computational methods, traditional machine learning and deep learning, are mainly used to predict ncRNA-protein interactions. Muppirals *et al.* proposed RPISeq, which is a computational model utilizing the information of sequence, encoding RNA and protein sequence through k-mers and classification through the SVM and Random Forest algorithms (Muppirala et al., 2011). RPI-SE method, developed by Yi *et al.*, extracts sequence information through k-mers sparse matrix and position weight matrix (PWM) with singular value decomposition (SVD) (Yi HC. et al, 2020). Suresh *et al.* designed model of RPI-Pred, same to RPISeq, which exploited RNA and protein sequence information and classified through SVM (Suresh et al., 2015). Wang *et al.* has developed an approach to make prediction of RNA-protein interactions based on sequence characteristics and naive Bayes classifier (Wang et al., 2013). catPAPID is introduced by Bellucci *et al.*, to exploit the physicochemical properties on nucleotide and polypeptide, and further to predict protein interactions in Xist network through catPAPID (Bellucci et al., 2011; Agostini et al., 2013). Cirillo *et al.* proposed method to predict protein-RNA interactions with Global Score, integrating local structure feature of RNA and protein into overall binding tendency, and calibrating through high-throughput data (Cirillo et al., 2017). Xiao *et al.* utilized the measure of HeteSim to score pairwise lncRNA-protein, and with the score, SVM was built to classify (Xiao et al., 2017). Li *et al.* applied LPIHN based on implementing random walk with restart on the heterogeneous network, including lncRNA-lncRNA similarity network, lncRNA-protein interactions network and protein-protein interaction network (Li et al., 2015). Methods proposed respectively by Zheng *et al.* and Yang *et al.* and the model of PLIPCOM extracted topological information of ncRNA-protein interactions by calculating the HeteSim scores on the relevance paths of the heterogeneous network (Yang et al., 2016; Zheng et al., 2017; Deng et al., 2018). Yao *et al.* used the knowledge graph with auto-encoder to detect protein complexes (Yao et al., 2020). DM-RPIs extracted sequence characteristics through making full use of stacked auto-encoder networks and trained through multiple base classifier (Cheng et al., 2019). NPI-RGCNAE is proposed by Yu *et al.* utilizing graph convolutional network (GCN) to predict ncRNA-protein interactions, and they developed a novel approach of negative sample selecting (Yu et al., 2021). Although existing computational methods using different RNA and protein features to predict with good performance, these methods may be ineffective due to the features may not available to all RNAs and proteins, particularly facing to new RNA and protein, which have no known interactions with any protein or RNA. Apart from that, existing approaches handled not good with long sequence and effective manner for feature extraction is crucial.

In this paper, to avoid existing deficiencies, we proposed a computational framework SAWRPI based on stacking ensemble. Traditional machine learning approaches have demonstrated their potential ability in small sample learning task, like prediction task of ncRNA-protein interactions with tree-based model and SVM (Yi H.-C. et al, 2020). Thus, our framework integrates four base classifiers XGBoost (Chen and Guestrin 2016), SVM (Cortes and Vapnik 1995; Chang and Lin 2011), ExtraTree (Geurts et al., 2006) and Random Forest (RF) (Breiman 2001) for classification and prediction. Specifically, we catch information of group-level amino acids through 3-mers sparse matrix, which contains the components of amino acid and the information of sequence order (You et al., 2016; Yi et al., 2019), and then generating feature vector through SVD. Meanwhile, method of natural language processing (NLP) is used to get representation of ncRNA nucleic acid symbols, then getting comprehensive information through a local fusion strategy. Next, Hilbert Transformation is exploited to further extract information and transform raw feature data to the new feature space which is easier to classify. Finally, inspired by *Pan et al.*(Pan et al., 2016), stacking ensemble is adopted to fuse all classification results from base predictors and generate final prediction results. To confirm the robustness and stability, three different datasets containing two kinds of interactions are utilized. When compared with state-of-the-art methods and other strategies for results classifying or feature extracting, our method achieved better performance. These results demonstrate the proposed framework is trustworthy and effective for ncRNA-protein interactions prediction.

## Materials and Methods

### Dataset Description

As the biological common sense, RNA contains two categories of mRNA and ncRNA. The ncRNA includes long non-coding RNA, which is longer than 200 nt, and small ncRNA, like miRNA and snoRNA and there are different biological functions among them (Pan et al., 2016). To demonstrate the robustness and stability of SAWRPI, different RNA-protein interactions benchmark datasets are used to validate, which including mRNA-protein and lncRNA-protein datasets. In practice, dataset RPI488 (Pan et al., 2016)and RPI369 (Muppiral et al., 2011), RPI1807 (Suresh et al., 2015) were chosen to evaluate. The first one is lncRNA-protein dataset, while the last two datasets stand for mRNA-protein. RPI488 is a non-redundant dataset of lncRNA-protein interactions, containing 245 negative samples and 243 positive samples among 25 lncRNAs and 247 proteins (Huang et al., 2010; Puton et al., 2012). Dataset RPI369 also is non-redundant with 332 RNA chains and 338 protein chains, generated from RPIDB (Lewis et al., 2010), a comprehensive database calculated from PDB (Berman et al., 2000), and has no ribosomal protein or ribosomal RNAs. It contains a total of 369 positive interactive pairs. RPI1807, a non-redundant dataset, generated by NDB (Lu et al., 2013), includes 1,078 RNAs and 1807 proteins, and then consist 1807 pairwise positive samples and 1,436 pairwise negative samples. Table 1 illustrates details of these three benchmark datasets.

**TABLE 1:** The details of the ncRNA-protein interactions datasets.

Data set	Interaction pairs	# of ncRNAs	# of proteins
RPI369	369	332	338
RPI1807	1807	1078	1807
RPI488	243	247	25

### Overview of Methods

**FIGURE 1:**
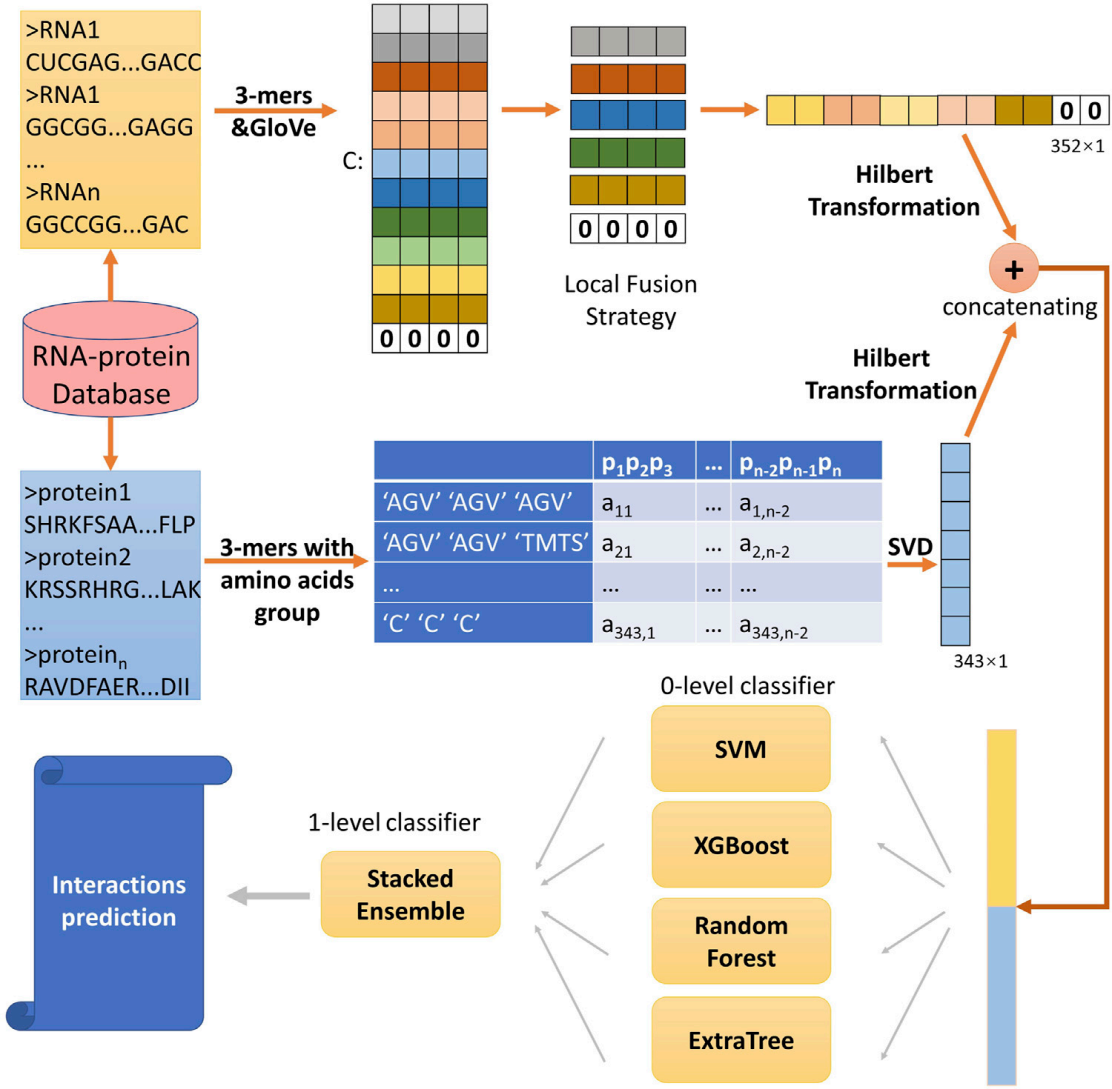
Pipeline of the framework of SAWRPI.

In this study, to predict ncRNA-protein interactions, we developed a computational method SAWRPI. Due to the difference of structure between ncRNA and protein, we extracted sequence information of two entities through different ways. For proteins, extracting conjoint triad (3-mers) from 7 groups of amino acids and generating 3-mers sparse matrix. Immediately, SVD is utilized to reduce the sparse matrix into a vector, which is seen as raw features. For ncRNA, word embedding method is used to extract raw representation of ncRNA symbol with the local fusion strategy (LFS). Before predicting through the classification strategy, Hilbert Transformation (HT) is used to further extract information of raw features. Finally, making prediction through the classifier with our strategy of stacking ensemble with adaptive weight initialization. Figure 1 deploys the detail of this process.

### Representation of ncRNA and Protein Sequences

To preliminarily obtain raw features, for each protein sequence, 20 amino acids are partitioned into 7 groups (Pan et al., 2010), “AGV”, “TMTS”, “ILFP”, “HNQW”, “DE”, “RK” and “C”, based on the dipole moments and side chain volume. Protein sequence with length of *n*, can be expressed using only seven symbols, and under sequence dividing into *n*-(*k*-1) subsequences, there are 7^*k*^ different possible *k*-mer. Then the *k* is set to 3 which is commonly accepted as empirical parameter (Shen et al., 2007; Yi et al., 2018). As Table 2 shown, the features of conjoint triad *p*_*j*_ *p*_*j+1*_ *p*_*j+2*_ based on the seven groups for each protein can be extracted as a sparse matrix *L*_*p*_ with the dimension of 7^*k*^×(*n*-(*k*-1)) (You et al., 2016), which can be defined as follows:

$$L_{p}=\left(a_{ij}\right),i\in \left[0,7^{k}-1\right],j\in \left[0,\left(n-\left(k-1\right)\right)\right]$$ (1)

$$a_{ij}=\left\{ \begin{array}{l} 1,\text{ if }p_{j}p_{j+1}p_{j+2}\;=\;k-mer\left(i\right)\\ 0,\text{ else} \end{array}\right.$$ (2)

**TABLE 2:** 3-mer sparse matrix of protein sequence.

	*p*_*1*_ *p*_*2*_ *p*_*3*_	*p*_*2*_ *p*_*3*_ *p*_*4*_	…	*p*_*n-2*_ *p*_*n-1*_ *p*_*n*_
‘AGV’ ‘AGV’ ‘AGV’	a_11_	a_12_	…	a_1,n-2_
‘AGV’ ‘AGV’ ‘TMTS’	a_21_	a_22_	…	a_2,n-2_
‘AGV’ ‘TMTS’ ‘AGV’	a_31_	a_32_	…	a_3,n-2_
…	…	…	…	…
‘C’ ‘C’ ‘C’	a_343,1_	a_343,2_	…	a_343,n-2_

Furthermore, the SVD is used to extract the vector with dimension of 7^*k*^×1 from sparse matrix *L*_*p*_. While, for each ncRNA sequence with length of *m*, *k*-mer composition is also used to divide them into *m*-(*k*-1) subsequences and the semantic information is utilized, which is different from the treatment processes of protein sequences. Each ncRNA can be considered as “sentence” and the subsequences (e.g., AAA, AAC, … , UUU) can be seen as “word”. Word embedding techniques have demonstrated the promise in natural language processing applications. Therefore, we used this technique to encode each subsequence. Specifically, features of global word co-occurrence probability are extracted through model of GloVe (Pennington et al., 2014), the details following the next section. Each “word” can be expressed as a feature vector, and each sentence with length of *m*-(*k*-1) are expressed as a feature matrix with dimension of *d*×(*m*-(*k*-1)), where *d* stands for dimension of embedding and is set to 32 in this experiment.

**FIGURE 2:**
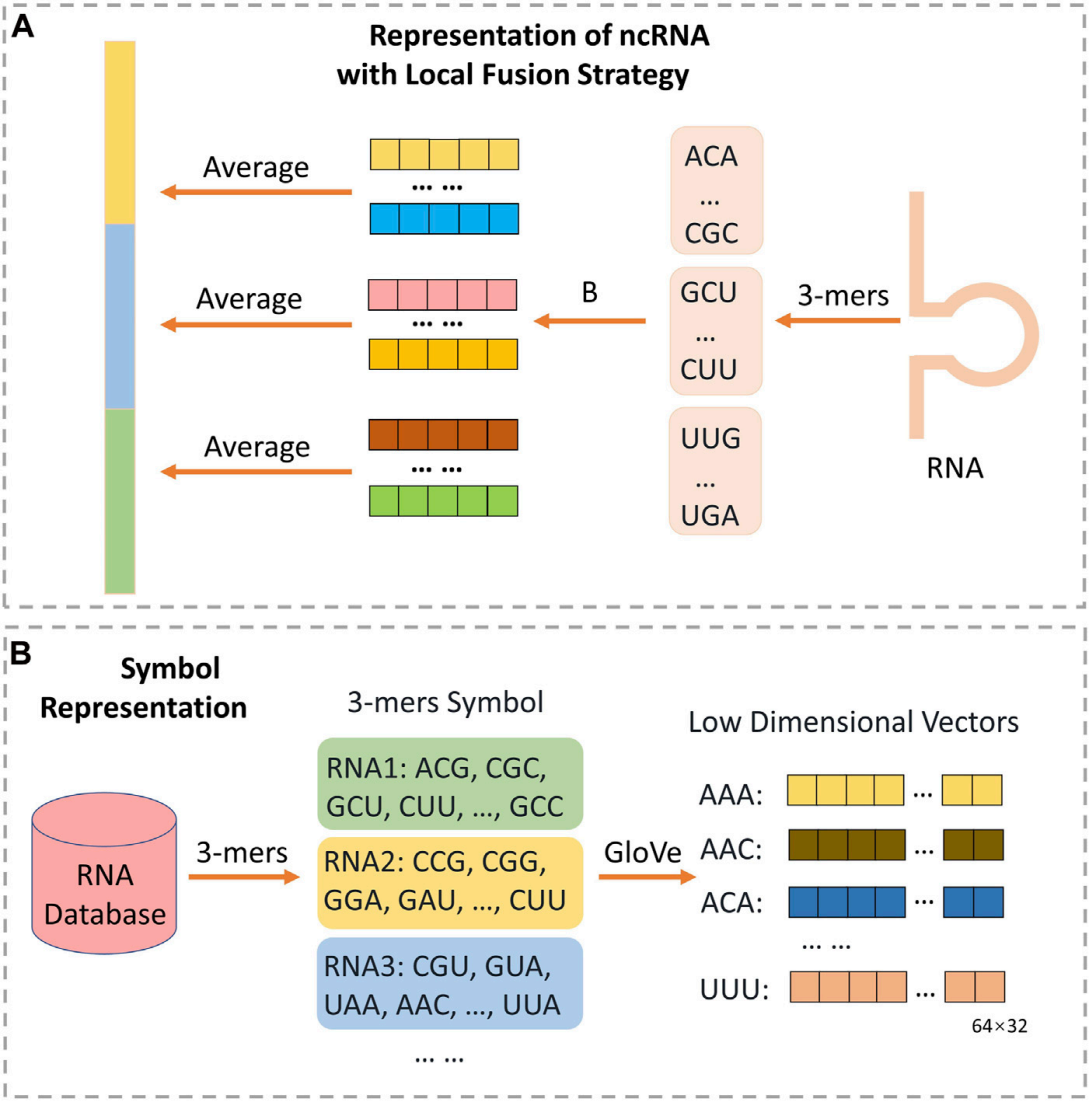
The architecture for extracting ncRNA structure feature through NLP method with local fusion strategy. As **(B)** shown, each ncRNA from database is divided into many triple symbols by 3-mers composition, and GloVe is used to generate embedding vector of 4^3^ symbols. Then, as **(A)** shown, each ncRNA will be split into some consecutive subsequences with no overlap. All the triple symbols embedding vector of each subsequence can be obtained from **(B)**. Finally, the representation of ncRNA can be obtained through calculating the average of all symbol vectors in each subsequence respectively, and concatenating all average vectors.

For long non-code RNA, there are more than 200-(*k*-1) words to be embedded. The count of feature factors is a tremendous overwhelming number. To solve it, many methods select the way of directly truncate, which is helpful but may loss many information of sequence (You et al., 2018; Chen et al., 2019; Yi H.-C. et al, 2020). Inspired by*.* Zeng et al. (2021) and motivated by spatial pyramid pooling-net (He et al., 2015), we proposed a novel local fusion strategy named LFS to fully explore the evolutionary features that after subsequence embedding, as Figure 2 shown, an average pooling layer is used to produce the patterns of the subsequence, and then combining all the pattern to a vector with certain dimension. Notably, if the length of RNA is too short to satisfy the setting dimension, zero will be filled. Finally, the raw feature vectors of each ncRNA and protein sequence can be extracted. And we set the number of groups as 11.

### Method of Word Embedding

One reason of deep learning technology developing rapidly is remarkably disposing of corpora in various fields. There are now many natural language processing methods and word embedding methods having been adopted, like iDeepSubMito (Hou et al., 2021), iCircRBP-DHN(Yang et al., 2021), Latent Semantic Analysis (LSA) (Dumais 2004), word2vec (Mikolov, et al., 2013; Mikolov, et al., 2013) and Global Vectors for Word Representation (GloVe) (Pennington et al., 2014). While in this paper, we exploit the model of GloVe to learning the embedding vectors of ncRNA “words”.

The model of GloVe can overcome the drawback of first two embedding methods mentioned previously that the high computational burden and utilization of partial corpus. It produces a word vector space, which has meaningful substructure, based on making full use of the information of global word-word co-occurrence. In detail, implementation of the GloVe is in a three-steps procedure. Firstly, constructing a co-occurrence matrix *X* based on ncRNA “word” corpus. Each co-occurrence matrix element *p*_*ij*_ stands for probability of co-occurrence rather than count of co-occurrence, following the formula:

$$p_{ij}=P\left(j|i\right)=\frac{x_{ij}}{x_{i}}$$ (3)

where *x*_*ij*_ represents for the appearing number of word *j* in the context environment of word *i*, and *x*_*i*_ stands for the total appearing number of all word in the context environment of the word *i*. Then, generating the word vector to construct approximation relationship with the co-ocurrence matrix through the function as follows.

$$\omega_{i}^{⊤}{\tilde{\omega }}_{j}+b_{i}+{\tilde{b}}_{j}=\log\left(x_{ij}\right)$$ (4)

where $\omega_{i}$ and ${\tilde{\omega }}_{j}$ respectively mean the embedding vectors of word *i* and word *j*, while $b_{i}$ and ${\tilde{b}}_{j}$ respectively mean bias terms. In the end, obtaining and minimizing the loss function:

$$J=\sum_{i,j=1}^{V}f\left(x_{ij}\right){\left(\omega_{i}^{⊤}{\tilde{\omega }}_{j}+b_{i}+{\tilde{b}}_{j}-\log\left(x_{ij}\right)\right)}^{2}$$ (5)

$$f\left(x\right)=\left\{ \begin{array}{l} {\left(x/x_{\max}\right)}^{\alpha }\text{ if }x<x_{\max}\\ 1\text{ otherwise} \end{array}\right.$$ (6)

where the f(⋅) is a weight function used to make the value of appearing number between the words rarely appearing much lower. In the experiment, we set embedding dimension as 32. After splitting nucleic acids sequences into 3-mers, each “words” can be indicated as a vector.

### Feature Extraction Method of Hilbert Transformation

To fully exploit sequence information, we further extract information from raw features. Hilbert transform (Johansson 1999) is used to generate features easily analyzing based on the raw features of ncRNA and protein. Hilbert transformation is usually used to analyze signal in the time and frequency, which acts as a 90° phase shifter without changing energy and amplitude, phase-shifting −90° to part of positive frequency, while phase-shifting 90° to part of negative frequency, and it can also be used as a tool of features extracting in the field of biology (Pan et al., 2021). The transformation function can be defined as:

$$\hat{x}\left(t\right)=x\left(t\right)\frac{1}{\pi t}=\frac{1}{\pi }\int_{-\infty }^{\infty }\frac{x\left(\tau \right)}{t-\tau }d\tau =-\frac{1}{\tau }\int_{-\infty }^{\infty }\frac{x\left(t+\tau \right)}{\tau }d\tau$$ (7)

where *x*(*t*) is seen as each feature vectors. And the back-transformation is defined as:

$$x\left(t\right)=-\hat{x}\left(t\right)\frac{1}{\pi t}=-\frac{1}{\pi }\int_{-\infty }^{\infty }\frac{\hat{x}\left(\tau \right)}{t-\tau }d\tau =\frac{1}{\tau }\int_{-\infty }^{\infty }\frac{\hat{x}\left(t+\tau \right)}{\tau }d\tau$$ (8)

Specifically, in this work, we respectively used model of SVD and GloVe to obtain the raw feature of protein and ncRNA. Then each protein and ncRNA is encoded as vectors with dimension of 7 × 7 × 7 and dimension of 11 × 32. Finally, after the processing of Hilbert transforming, hidden high-level features can be extracted.

### Machine Learning Base Classifier

In this work, four kinds of machine learning base classifiers are utilized to integrate, including XGBoost (Chen and Guestrin 2016), SVM (Cortes and Vapnik 1995; Chang and Lin 2011), ExtraTree (Geurts et al., 2006) and Random Forest (Breiman 2001). SVM is used for classification, regression or other work, through constructing one or multiple hyperplanes in a high-dimension space. Intuitively, a decent segmentation using the hyperplane can maximize the distance of function margins (points of training data) in any class. It is usually used in high dimension space with high-performance, although the sample size is lower than data dimension. However, if the number of samples is much lower than the number of the data features, SVM may overfitting and need to select efficient kernel to avoid.

Supposing the training dataset with label [(*x*_*i*_ *, y*_*i*_), *i* = 0, 1, … , *n*, *y*_*i*_ = (1, -1), *x*_*i*_∈ R] and regarding (*w*(*x*)+*b*) = 0 as a separating hyperplane. In the linear separable problems, to maximize the margin, SVM minimizes subject of ||*w*||^2^/2 to find the separation hyperplane through the constraint:

$$y_{i}\left(w_{x_{i}}+b\right)\geq 1,\forall x_{i}$$ (9)

And in the linear non-separable problems, slack variables are introduced to look for the optimal separating hyperplane, then minimizing the function:

$$||w||2/2+C\sum_{i=1}^{n}\xi_{i},\xi_{i}\geq 0,\forall x_{i}$$ (10)

$$y_{i}\left(w_{x_{i}}+b\right)\geq 1-\xi_{i},\xi_{i}\geq 0,\forall x_{i}$$ (11)

where *C* is user-adjustable parameter. Kernel of Radial Basis Function (RBF) is adopted, which is defined as:

$$f\left(x\right)=e^{-\gamma ||x-x'|{|}^{2}}$$ (12)

XGBoost, a model of end-to-end tree boosting, can perceive sparsity data well called sparsity-aware. To control complexity of the model, XGBoost adds a regularization term to cost function, which can reduce the variance of the model as well as prevent situation of overfitting, and then performs second-order Taylor expansion. For a larger learning space, XGBoost diminishes the impact of each tree through multiplying the weight of leaf nodes. Its objective function is defined as follows.

$$Obj=\sum_{i=1}^{n}l\left(y_{i},{\hat{y}}_{i}\right)+\sum_{k=1}^{K}\Omega \left(f_{k}\right)$$ (13)

$$\Omega \left(f_{t}\right)=\gamma T+\frac{\lambda }{2}\sum_{j=1}^{T}w_{j}^{2}$$ (14)

where *l* is used to compute difference between target $y_{i}$ and prediction ${\hat{y}}_{i}$ . Then, Ω(⋅) stands for regular term containing *T*, count of leaf nodes, and the sum of *l*_2_ modulus square of score on each leaf. XGBoost supports column sampling and draws on the method of Random Forest, which can avoid over-fitting and save computation resources.

Random Forest is a representative ensemble classification algorithm, which is based on the decision tree evaluator to introduce randomness features selection into the process of decision tree training. Specifically, it uses multiple decision tree to reduce variance of output. For each node of decision tree, randomly selecting a subset containing *K* features from the node features set, and then optimal features can be selected from subset to split. The *K* is used to control degree of randomness. Supposing the label sets is $\left\{c_{1},c_{2},...,c_{N}\right\}$ and the prediction of *i*th base classifier on the sample is ${\left(h_{i}^{1}\left(x\right),h_{i}^{2}\left(x\right),...,h_{i}^{N}\left(x\right)\right)}^{⊤}$ . For integrating results of each base classifier, majority voting and averaging methods are often used, which are respectively defined as:

$$H\left(x\right)=\left\{ \begin{array}{l} c_{j},\;\sum_{i=1}^{T}h_{i}^{j}\left(x\right)>0.5+\sum_{k=1}^{N}\sum_{i=1}^{T}h_{i}^{k}\left(x\right)\\ reject,\;otherwise \end{array}\right.$$ (15)

$$H\left(x\right)=\frac{1}{T}\sum_{i=1}^{T}w_{i}h_{i}\left(x\right)$$ (16)

where *w*_*i*_ is weight of *i*th base classifier. Extremely randomized tree (ExtraTree) is on the basis of random forest to further random on splitting threshold. And extremely randomized tree essentially builds totally randomized trees, which selects attribute and cut-point with strongly randomizing when it splits a tree node. Tree structure is independent of the output value. It can further enhance randomness of segmentation points that choosing suitable parameter according specific task. Under the segmentation rule, selecting the best threshold for each candidate feature from these randomly generated thresholds.

And all the parameters were set as follows. The sklearn tool was used in this paper to training four models. For the parameters of XGBoost, we set max_depth = 6 and booster = 'gblinear’. The kernel of ‘rbf’ is set for SVM model. There are four parameters to Random Forest model, criterion = 'gini’, n_estimators = 25, random_state = 1 and n_jobs = 2. Model of ExtraTree uses default parameters.

### Strategy of Stacking Ensemble With Adaptive Weight Initialization

Ensemble learning method accomplished learning task through constructing and combining multiple evaluators rather than one learning machine, which considers multiple results of each evaluator and integrates into a comprehensive result. In most situations, multiple evaluators are better than single evaluators in performance of classification and regression task.

**FIGURE 3:**
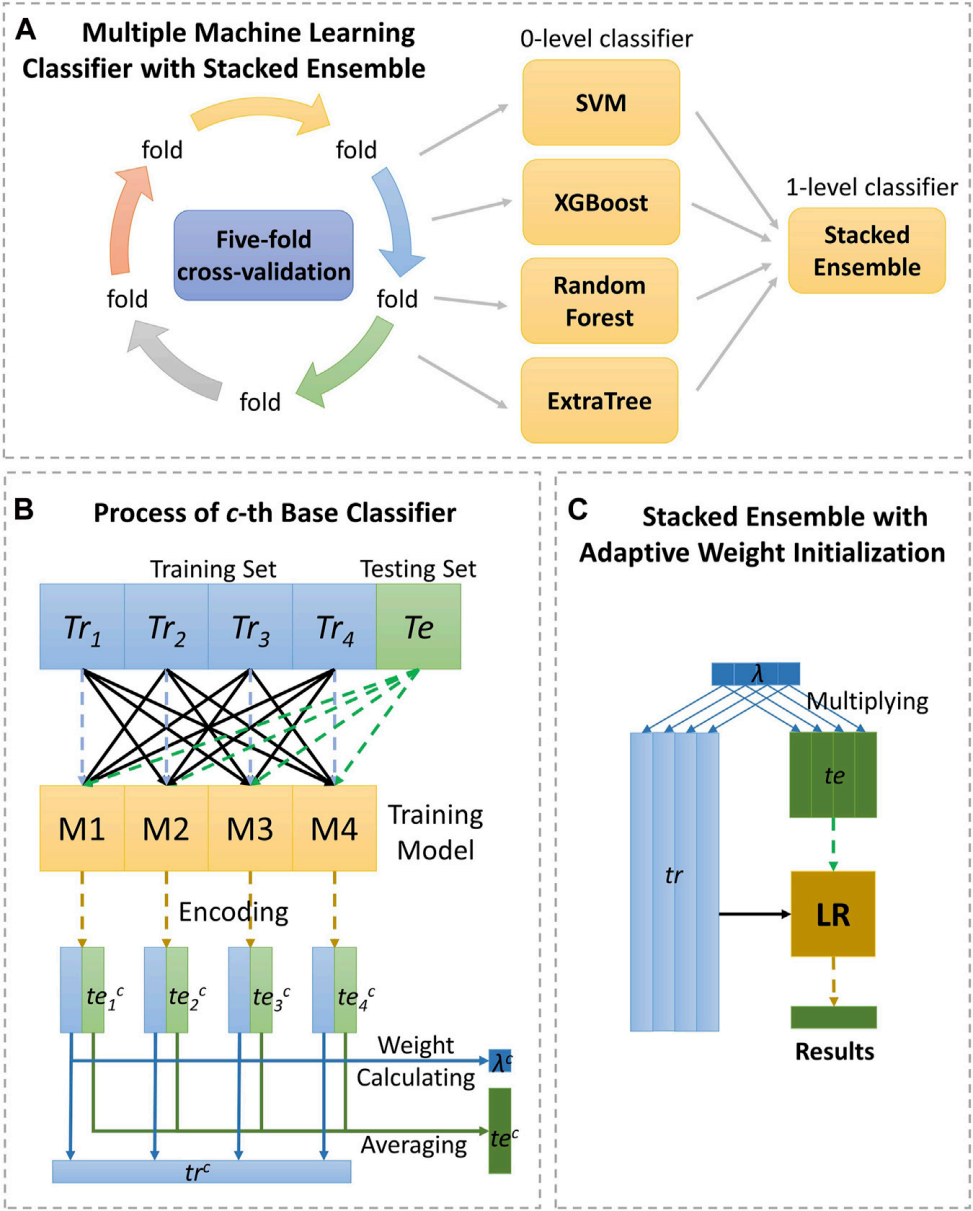
The detailed process of the strategy, stacking ensemble with adaptive weight initialization. As **(A)** shown, the data are calculated by the four classifiers under five-fold cross-validation, respectively and making final prediction through stacked ensemble strategy. Section **(B)** displays the process of 0-level classifier, and section **(C)** displays the process of 1-level classifier.

Generally, different performances are present in different classifiers (evaluators). And how to efficiently integrate different classifiers to generate the target function is so crucial. Previously, there are many studies of integrating multiple classifiers, containing majority voting (Breiman 2001), averaging results of each base model (Pan et al., 2011) and stacked ensemble method (Töscher et al., 2009). Majority voting and averaging has been detailed previously. While, stacked ensembling follows the intuition of the deep neural network, uniting with encoder layer and successive decoder layer. Specifically, the level 0 classifiers, regarded as encoder layer, firstly generate prediction probability score, and then, the level 1 classifier integrate results from single classifier through logistic regression. Figure 3 shows the detail as follows.

In the encoding layer with *c*th base classifier, the training set *Tr* will be split divided into four equal fractions *Tr*_*i*_ and encoded in four runs. In *i*th run, training sub-set of *Tr*_*i*_ is encoded by the sub-encoder learning from the rest of the training sub-sets through *c*th base classifier, and the testing set *Te* also is encoded as a vector of *te*_*i*_^*c*^. After four iterations, with *c*th classifier, the training set *Tr* can be expressed in *tr* ^*c*^, and the testing set *Te* can be expressed in *te* ^*c*^ through the function as follows:

$$te^{c}=\frac{1}{N}\sum_{i=1}^{N}te_{i}^{c}$$ (17)

where *N* means the number of base classifiers. Through all of the base classifiers, encoding matrix of *Tr* and *Te* can be generated, whose rows stand for encoding vectors of all the samples. Then, level 1 layer of logistic regression satisfies the following equations:

$$P_{w}\left(y=\pm 1|x\right)=\frac{1}{1+e^{-yw^{⊤}x}}$$ (18)

where *x* is encoding vector, and *w* is learning weight vector for each classifier. When *w* is same constant for each classifier, it is equivalent to strategy of averaging, however, if only one element of is non-zero, it is like strategy of majority voting.

In this work, we provided a strategy of adaptive weight initialization through initialization parameter *λ* ^*c*^ for *c*th classifier which is defined as follows.

$$\lambda^{c}=-\frac{1}{\left(1-\frac{1}{{\left(w^{c}\right)}^{2}}\right)N}$$ (19)

$$w^{c}=\frac{1}{N}\sum_{i=1}^{N}w_{i}^{c}$$ (20)

where *w*_*i*_^*c*^ stands for the AUC score of *Tr*_*i*_ prediction with *c*th classifier in each run mentioned above. The aim of arising parameter *λ* ^*c*^ is making the importance of weaker classifier to reduce before feeding the vectors to decoder layer to improve performance by fine-tuning. Thus, *Tr* and *Te* can be expressed in *λ* ^*c*^×*tr* ^*c*^ and *λ* ^*c*^×*te* ^*c*^ respectively with *c*th classifier.

## Experimental Results and Discussion

### Evaluation Criteria

In this article, the performance of SAWRPI is evaluated by five-fold cross validation. And each validation makes full use of the frequently utilized metrics to assess robustness and effectiveness of the proposed method. Including Accuracy (Acc.), Sensitivity (Sen.), Precision (Prec.), F1 (Macro F1) and MCC (Matthews’s Correlation Coefficient). These evaluation indicators can be represented as follows:

$$Acc.=\frac{TP+TN}{TN+TP+FN+FP}$$ (21)

$$Prec.=\frac{TP}{TP+FP}$$ (22)

$$Sen.=\frac{TP}{TP+FN}$$ (23)

$$F1=\frac{2\times Prec.\times Sen.}{Prec.+Sen.}$$ (24)

$$MCC=\frac{TP\times TN-FP\times FN}{\sqrt{\left(TP+FP\right)\times \left(TN+FN\right)\times \left(TN+FP\right)\times \left(TP+FN\right)}}$$ (25)

where TP and FN are treated as the number of positive samples which are correctly predicted as positive and incorrectly predicted as negative, respectively, then TN and FP respectively stand for the number of negative samples which are correctly detected as negative and incorrectly detected as positive. Apart from the above indicators, AUC, the area under the ROC curves, is constructed to evaluate our model. The mean value of the results of five validation is used to ensure low-variance and unbiased evaluations.

### Assessment of Prediction Ability

In this work, to demonstrate performance and robustness of SAWRPI, three datasets, indicating two kinds of ncRNA-protein interactions, have been used to validate, including mRNA-protein and lncRNA-protein datasets. Furthermore, the five-fold cross-validation can enhance the persuasion of the predicting results. Specifically, dataset RPI369, RPI488 and RPI1807 is used to evaluate SAWRPI. Table 3 reveals the result of prediction. Certainly, the same experiments with the other classifiers are reported in Supplementary Material.

**Table 3:** Five-Fold cross-validation results on three datasets by SAWRPI.

Dataset	Fold	Acc	Prec	Sen	F1	MCC
RPI369	0	0.743	0.720	0.797	0.756	0.489
	1	0.682	0.667	0.730	0.697	0.367
	2	0.696	0.688	0.716	0.702	0.392
	3	0.721	0.709	0.757	0.732	0.443
	4	0.707	0.679	0.781	0.726	0.420
	**Average**	**0.710 ± 0.023**	**0.693 ± 0.022**	**0.756 ± 0.034**	**0.723 ± 0.024**	**0.422 ± 0.047**
RPI488	0	0.918	0.976	0.851	0.909	0.842
	1	0.897	0.972	0.795	0.875	0.800
	2	0.876	0.911	0.879	0.895	0.746
	3	0.918	0.955	0.875	0.913	0.838
	4	0.866	0.878	0.818	0.847	0.729
	**Average**	**0.895 ± 0.024**	**0.938 ± 0.042**	**0.844 ± 0.036**	**0.888 ± 0.027**	**0.791 ± 0.052**
RPI1807	0	0.963	0.954	0.981	0.967	0.925
	1	0.969	0.965	0.981	0.973	0.938
	2	0.963	0.957	0.978	0.967	0.925
	3	0.966	0.964	0.975	0.970	0.931
	4	0.975	0.967	0.989	0.978	0.950
	**Average**	**0.967 ± 0.005**	**0.961 ± 0.006**	**0.981 ± 0.005**	**0.971 ± 0.004**	**0.934 ± 0.011**

As the table shown, the average scores of Acc reach 0.710, 0.895, and 0.967 in all three datasets. When applying SAWRPI to RPI1807, we obtained the highest average score of Acc, Prec, Sen, F1 and MCC of 0.967, 0.961, 0.981, 0.971, and 0.934, with the standard deviation of 0.005, 0.006, 0.005, 0.004, and 0.011, respectively. On the dataset of RPI369, whose type of interaction is same to RPI1807, obtained average Acc, Prec, Sen, F1 and MCC of 0.710, 0.693, 0.756, 0.723 and 0.422, with the standard deviation of 0.023, 0.022, 0.034, 0.024 and 0.047, respectively. Comparing these results, it is easy to see that SAWRPI is more applicable to the dataset of RPI1807. Thus, the size of dataset can cause effect on prediction result. The other type dataset RPI488 reached average Acc, Prec, Sen, F1 and MCC of 0.895, 0.938, 0.844, 0.888 and 0.791, with the standard deviation of 0.024, 0.042, 0.036, 0.027 and 0.052, respectively. At the view of interaction type, our model may be more effective on the interaction type of lncRNA-protein. One reason may be that our method of representing ncRNA can capture more distal sequence information, which may bring some noise at the same time. Even then, it is undeniable that SAWRPI still achieved a fabulous capability of ncRNA-protein interactions prediction.

### Comparison Between Different Classification Strategies

AUC, the area under ROC curve, is regarded as an important criterion for evaluating the performance of the classification model. To verify the superiority of our strategy of stacking ensemble with adaptive weight initialization, we compared it with two different integrating methods in the same features of ncRNA and protein. As Table 4 shown, our integrating strategy is more advantageous on dataset of RPI369 and RPI488, and competitive on dataset of RPI1807. The results of other evaluation parameters are reported in Supplementary Material.

**TABLE 4:** AUC of different integrating methods on three datasets.

Integrating method	RPI369	RPI488	RPI1807
Averaging	0.737	0.919	0.993
Ensemble	0.744	0.921	0.992
Ensemble with initialization	0.746	0.922	0.992

The bold values represent the higher values each column.

**FIGURE 4:**
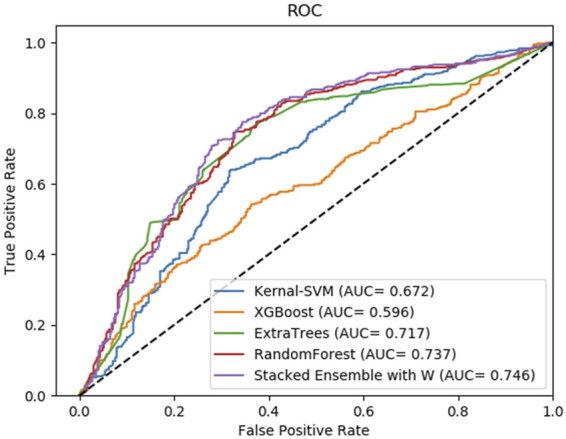
Average result of ROC curves of five-fold cross-validation with four single base classifiers and our method of stacking ensemble on RPI369 by SAWRPI. AUC expresses area under an ROC curve.

**FIGURE 5:**
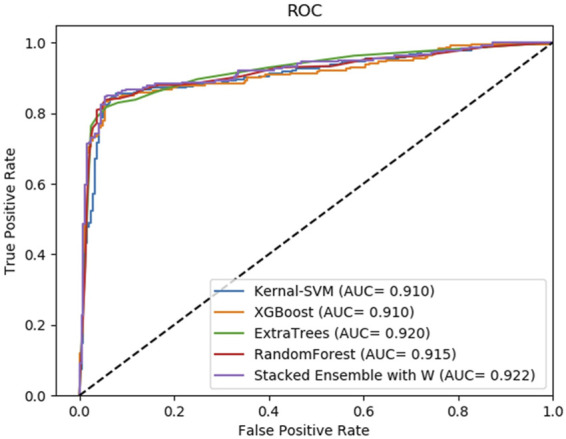
Average result of ROC curves of five-fold cross-validation with four single base classifiers and our method of stacking ensemble on RPI488 by SAWRPI. AUC expresses area under an ROC curve.

**FIGURE 6:**
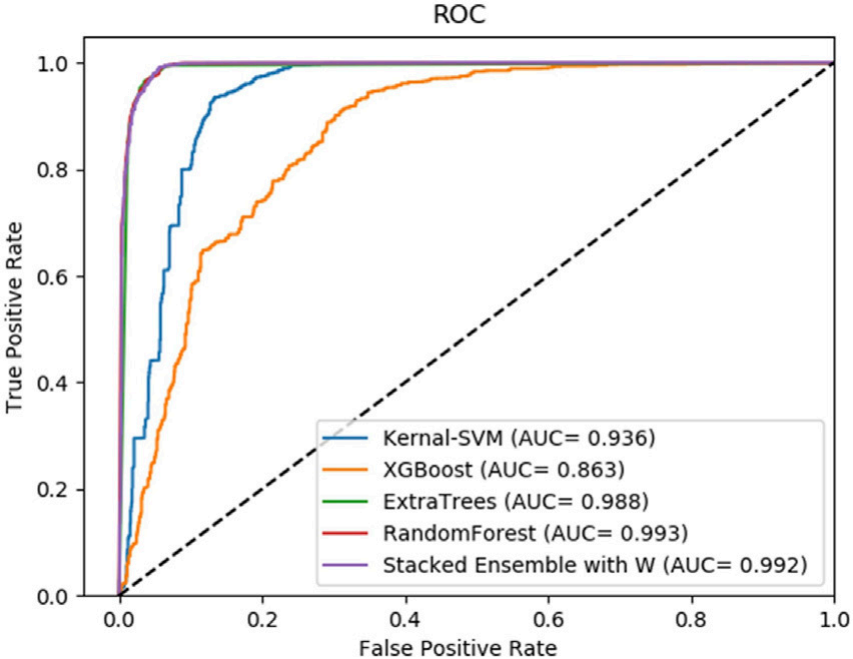
Average result of ROC curves of five-fold cross-validation with four single base classifiers and our method of stacking ensemble on RPI1807 by SAWRPI. AUC expresses area under an ROC curve.

**FIGURE 7:**
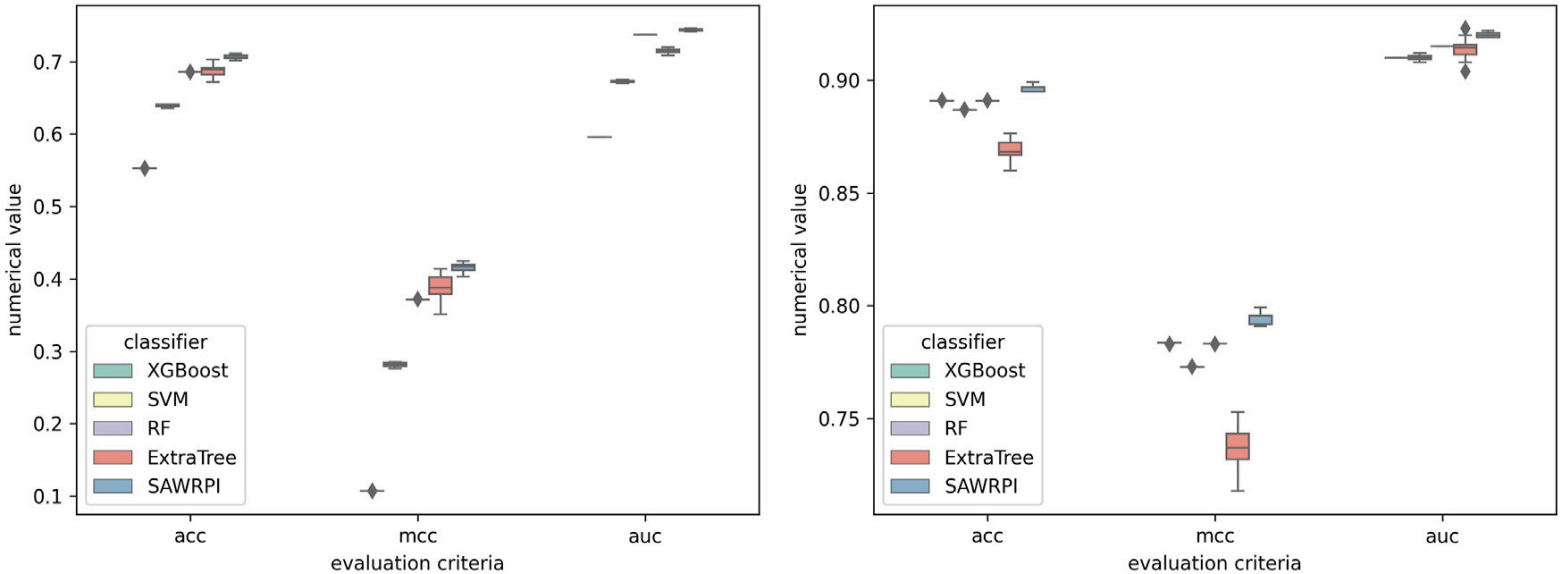
Experimental results of SAWRPI on RPI369 and RPI488 datasets with different classifiers. The result of SAWRPI on RPI1807 is shown in Supplementary Material.

Moreover, to reveal the improvement of stacking ensemble strategy, we also contrasted our strategy with the four classifiers, which are used as base predictors of our method. Integrating four base predictors through a Logistic Regression function automatically. As Table 5 illustrates, on the RPI369 dataset, SAWRPI obtained five the highest values of Acc, Prec, Sen, F1 and MCC of 0.710, 0.692, 0.756, 0.723 and 0.422, respectively. On the RPI488 dataset, SAWRPI got four the highest values of Acc, Prec, F1 and MCC of 0.895, 0.938, 0.888 and 0.791, respectively. On the RPI1807 dataset, SAWRPI obtained four the highest values of Acc, Sen, F1 and MCC of 0.967, 0.981, 0.971 and 0.934, respectively. Although the results of our method are not the best on each criterion, it still obtained comparable results which are only 0.004 and 0.005 lower than the best value, respectively. For further description of the model reliability, three ROC curves displayed following, shown by Figures 4–6. To verify that the results are truly significant, statistical learning method is used to plot boxplots, shown by Figure 7. Additionally, ROC curves figures of comparing all classifying strategies in three datasets and five-fold cross-validation results on three datasets by different classifying strategies are shown in Supplementary Material.

**TABLE 5:** Five-Fold cross-validation average results on three datasets by different classifiers.

Dataset	Classifier	Acc	Prec	Sen	F1	MCC
RPI369	XGBoost	0.553	0.551	0.596	0.571	0.107
	SVM	0.638	0.661	0.569	0.610	0.280
	RF	0.686	0.685	0.686	0.685	0.372
	ExtraTree	0.690	0.677	0.726	0.700	0.381
	**SAWRPI**	**0.710**	**0.692**	**0.756**	**0.723**	**0.422**
RPI488	XGBoost	0.891	0.941	0.831	0.882	0.783
	SVM	0.887	0.916	**0.848**	0.880	0.773
	RF	0.891	0.935	0.837	0.883	0.783
	ExtraTree	0.860	0.877	0.837	0.855	0.720
	**SAWRPI**	**0.895**	**0.938**	0.844	**0.888**	**0.791**
RPI1807	XGBoost	0.802	0.754	0.959	0.844	0.617
	SVM	0.899	0.876	0.952	0.913	0.796
	RF	0.965	**0.966**	0.971	0.969	0.929
	ExtraTree	0.965	0.960	0.978	0.969	0.930
	**SAWRPI**	**0.967**	0.961	**0.981**	**0.971**	**0.934**

The bold values represent the higher values each column of each dataset.

### Comparison Between Different Feature Extracting Strategies

**FIGURE 8:**
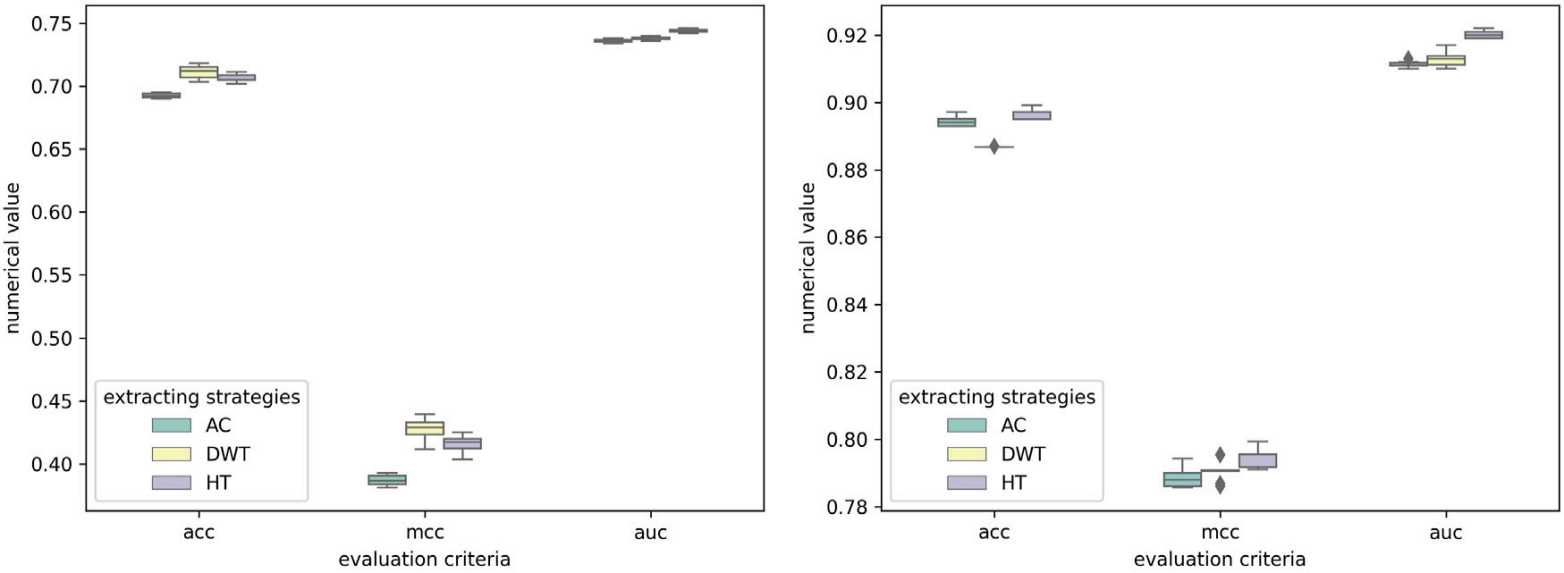
Experimental results of SAWRPI on RPI369 and RPI488 datasets with different feature extracting strategies. The result of SAWRPI on RPI1807 is shown in Supplementary Material.

To illustrate the effectiveness of feature extraction method, HT was compared with some correlatively common methods, including Auto-covariance (AC) (Zeng et al., 2009) and Discrete Wavelet transform (DWT) (Nanni et al., 2012). As shown in Table 6, on the RPI369 and RPI1807 dataset, our method got the highest prediction values on all evaluation criteria of 0.710, 0.692, 0.756, 0.723, 0.422 and 0.746, and 0.967, 0.961, 0.981, 0.971, 0.934 and 0.992, respectively. And on the RPI488 dataset, our method obtained only 0.008 lower accuracy in term of Sen, comparing the highest value. Obviously, the performance of our feature extracting strategies is better than the others. To verify that the results are truly significant, statistical learning method is used to plot boxplots shown by Figure 8. Notably, the five-fold cross-validation results table and the ROC curve figures of each classification method mentioned above based on different feature extracting strategies are reported in the Supplementary Material.

**TABLE 6:** Five-Fold cross-validation average results on three feature extracting strategies.

Dataset	Strategies	Acc	Prec	Sen	F1	MCC	AUC
RPI369	AC	0.690	0.675	0.732	0.702	0.381	0.737
	DWT	0.706	0.689	0.751	0.718	0.414	0.736
	HT	**0.710**	**0.692**	**0.756**	**0.723**	**0.422**	**0.746**
RPI488	AC	0.893	0.923	**0.852**	0.886	0.786	0.910
	DWT	0.893	0.932	0.843	0.885	0.786	0.913
	HT	**0.895**	**0.938**	0.844	**0.888**	**0.791**	**0.922**
RPI1807	AC	0.961	0.960	0.971	0.965	0.921	0.992
	DWT	0.965	0.961	0.977	0.969	0.929	0.992
	HT	**0.967**	**0.961**	**0.981**	**0.971**	**0.934**	**0.992**

The bold values represent the higher values each column of three datasets.

### Comparison With Other State-of-The-Art Methods

Furthermore, in order to verify effectiveness and stability of SAWRPI, we compared SAWRPI with other state-of-the-art computational approaches in the same three datasets that RPI488, RPI369 and RPI 1807. The contrast methods include RPISeq-RF (Muppirala et al., 2011), lncPro(Lu et al., 2013), SDA-RF (Pan et al., 2016) and SDA-FT-RF (Pan et al., 2016), which are based on sequence information and similar to SAWRPI. The authors, proposing method of RPISeq-RF, also developed another method RPISeq-SVM to predict. We only used RPISeq-RF which has better performance as comparation. Comparison methods of SDA-RF and SDA-FT-RF respectively used stacked denoising autoencoder through RF classification and stacked denoising autoencoder with fine tuning through RF classification. Table 7 shows all of the results of comparison. Through comparing with any other methods, it can be indicated that a little better performance of our method with Acc of 0.710, Sen of 0.756, F1 of 0.723 and MCC of 0.422. For the RPI1807 dataset, SAWRPI also gives a good performance in Prec, Sen and F1 with 0.961, 0.987 and 0.971. On RPI369 and RPI1807 datasets, SAWRPI obtained acceptable performance and got the highest value in term of F1 with 0.723 and 0.971 respectively. For the lncRNA-protein interactions dataset RPI488, our method achieved significant dominance in the important parameter AUC with 0.922 and displayed the performance with the outstanding improvements of 0.025–0.015, 0.028–0.006, 0.051–0.029 and 0.021–0.013 against others in terms of Acc, Prec, MCC and AUC respectively. Proposed method got the highest result in multiple criteria on three datasets, and notably, the best results in terms of highest AUC were obtained on RPI488. This illustrates that our method has more obvious advantages in task of predicting lncRNA-protein interactions. Without a doubt, SAWRPI is a powerful method of predicting ncRNA-protein interactions.

**TABLE 7:** Results of comparing with state-of-the-art methods on three datasets.

Dataset	Method	Acc	Prec	Sen	F1	MCC	AUC
RPI369	RPISeq-RF	0.704	0.707	0.705	0.706	0.409	**0.767**
	lncPro	0.704	**0.713**	0.708	0.710	0.409	0.740
	SDA-RF	0.707	0.689	0.699	0.694	0.416	0.754
	SDA-FT-RF	0.693	0.602	0.664	0.631	0.396	0.728
	SAWRPI	**0.710**	0.692	**0.756**	**0.723**	**0.422**	0.746
RPI488	RPISeq-RF	0.880	0.932	**0.926**	**0.929**	0.762	0.903
	lncPro	0.870	0.910	0.900	0.905	0.740	0.901
	SDA-RF	0.880	0.928	0.922	0.925	0.762	0.904
	SDA-FT-RF	0.881	0.926	0.916	0.921	0.762	0.909
	SAWRPI	**0.895**	**0.938**	0.844	0.889	**0.791**	**0.922**
RPI1807	RPISeq-RF	**0.973**	0.960	0.968	0.964	**0.946**	**0.996**
	lncPro	0.969	0.955	0.965	0.960	0.938	0.994
	SDA-RF	0.972	0.962	0.970	0.966	0.944	0.995
	SDA-FT-RF	0.972	0.940	0.955	0.947	0.944	0.995
	SAWRPI	0.967	**0.961**	**0.981**	**0.971**	0.934	0.992

The bold values represent the higher values each column of three datasets.

## Conclusion

In this work, we provided a computational model named SAWRPI which can predict ncRNA-protein interactions utilizing sequence information through integrates four individual base classifiers, including SVM, XGBoost, ExtraTrees and Random Forest. LFS and k-mers sparse matrix with HT are made full use of extracting efficient feature. It is proven that SAWRPI can accurately predict potential ncRNA-protein interactions and get good performance on both of small and large datasets. Besides, comparative analysis of different classification strategies and different feature extracting strategies respectively demonstrated superior performance of our classification strategies and using HT to generate final features. Furthermore, comparing with state-of-the-art method indicates our method has advantages of predicting potential interactions, specifically on predicting ncRNA-protein interactions. There is no doubt that our method can provide a useful guidance for ncRNA-protein interactions related biomedical research. In the future, more effective feature extracting strategy and adding other biological information to the model may bring higher accuracy and improve the performance.

## Data Availability

The original contributions presented in the study are included in the article/Supplementary Material, further inquiries can be directed to the corresponding authors.
