# Supplementary material for: SAWRPI: A Stacking Ensemble Framework With Adaptive Weight for Predicting ncRNA-Protein Interactions Using Sequence Information
Source: Front Genet. 2022 Feb 28;13:839540. doi: 10.3389/fgene.2022.839540 (PMC8963817; doi:10.3389/fgene.2022.839540)
Supplement: Supplementary file 1 [file DataSheet1.docx]

Supplementary Material

# ROC curves figures of comparing all classifying strategies in three datasets


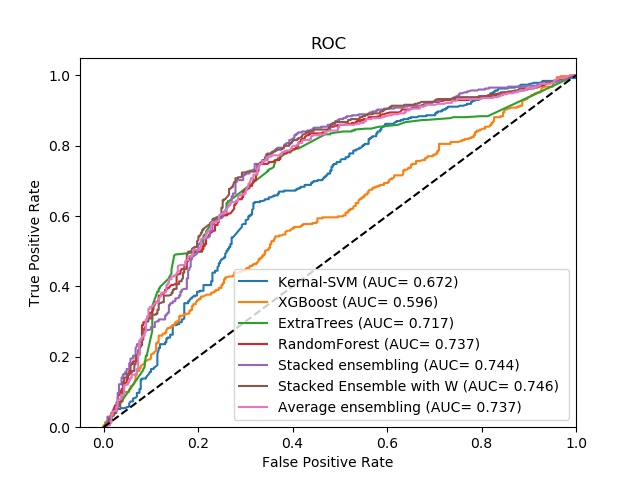


**Supplementary Figure 1.** ROC curves figures of comparing all classifying strategies in RPI369 datasets


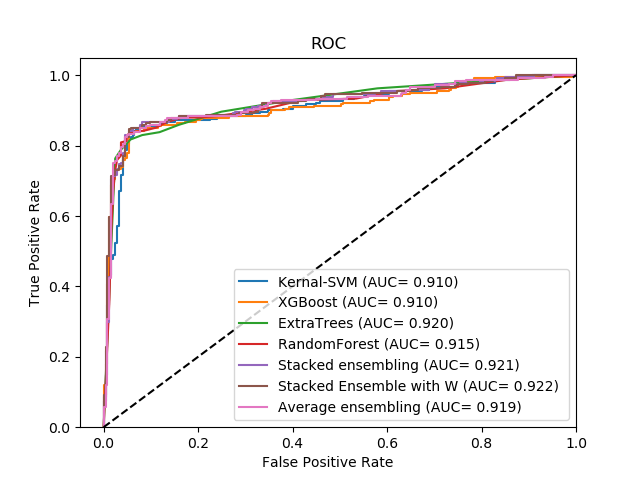


**Supplementary Figure 2.** ROC curves figures of comparing all classifying strategies in RPI488 datasets


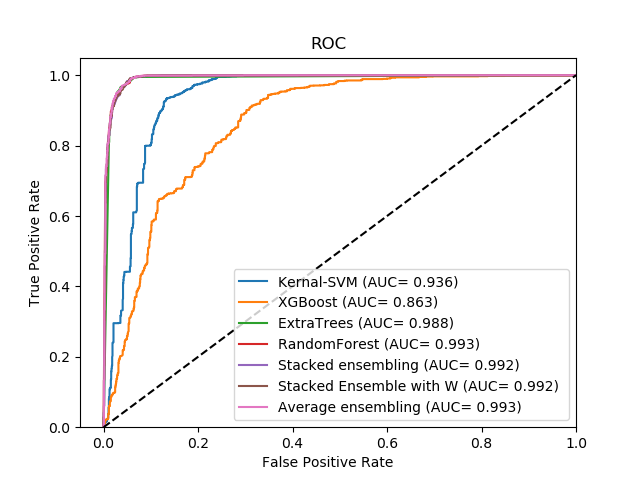


**Supplementary Figure 3.** ROC curves figures of comparing all classifying strategies in RPI1807 datasets

# Results of five-fold cross-validation on three datasets with each classifier

## SVM

Table 1. Five-Fold cross-validation results on RPI369 by SAWRPI with SVM

| **Fold** | **Acc.** | **Prec.** | **Sen.** | **F1** | **MCC** |
| --- | --- | --- | --- | --- | --- |
| 0 | 0.615 | 0.66 | 0.473 | 0.551 | 0.240 |
| 1 | 0.622 | 0.629 | 0.595 | 0.611 | 0.244 |
| 2 | 0.655 | 0.672 | 0.608 | 0.638 | 0.312 |
| 3 | 0.619 | 0.645 | 0.541 | 0.588 | 0.242 |
| 4 | 0.680 | 0.697 | 0.630 | 0.662 | 0.362 |
| **Average** | **0.638±0.028** | **0.661±0.026** | **0.570±0.063** | **0.610±0.043** | **0.280±0.055** |

Table 2. Five-Fold cross-validation results on RPI488 by SAWRPI with SVM

| **Fold** | **Acc.** | **Prec.** | **Sen.** | **F1** | **MCC** |
| --- | --- | --- | --- | --- | --- |
| 0 | 0.918 | 0.976 | 0.851 | 0.909 | 0.842 |
| 1 | 0.876 | 0.921 | 0.795 | 0.854 | 0.754 |
| 2 | 0.876 | 0.911 | 0.879 | 0.895 | 0.746 |
| 3 | 0.918 | 0.935 | 0.896 | 0.915 | 0.836 |
| 4 | 0.845 | 0.837 | 0.818 | 0.828 | 0.688 |
| **Average** | **0.887±0.031** | **0.916±0.051** | **0.848±0.042** | **0.880±0.038** | **0.773±0.065** |

Table 3. Five-Fold cross-validation results on RPI1807 by SAWRPI with SVM

| **Fold** | **Acc.** | **Prec.** | **Sen.** | **F1** | **MCC** |
| --- | --- | --- | --- | --- | --- |
| 0 | 0.888 | 0.862 | 0.950 | 0.904 | 0.774 |
| 1 | 0.917 | 0.887 | 0.975 | 0.929 | 0.835 |
| 2 | 0.900 | 0.874 | 0.958 | 0.914 | 0.800 |
| 3 | 0.887 | 0.871 | 0.936 | 0.903 | 0.772 |
| 4 | 0.901 | 0.888 | 0.942 | 0.914 | 0.800 |
| **Average** | **0.899±0.012** | **0.876±0.011** | **0.952±0.015** | **0.913±0.010** | **0.796±0.026** |

## XGBoost

Table 4. Five-Fold cross-validation results on RPI369 by SAWRPI with XGBoost

| **Fold** | **Acc.** | **Prec.** | **Sen.** | **F1** | **MCC** |
| --- | --- | --- | --- | --- | --- |
| 0 | 0.561 | 0.563 | 0.541 | 0.552 | 0.122 |
| 1 | 0.554 | 0.545 | 0.649 | 0.593 | 0.110 |
| 2 | 0.595 | 0.597 | 0.581 | 0.589 | 0.189 |
| 3 | 0.531 | 0.533 | 0.541 | 0.537 | 0.061 |
| 4 | 0.524 | 0.516 | 0.671 | 0.583 | 0.052 |
| **Average** | **0.553±0.028** | **0.551±0.031** | **0.597±0.061** | **0.571±0.025** | **0.107±0.055** |

Table 5. Five-Fold cross-validation results on RPI488 by SAWRPI with XGBoost

| **Fold** | **Acc.** | **Prec.** | **Sen.** | **F1** | **MCC** |
| --- | --- | --- | --- | --- | --- |
| 0 | 0.918 | 0.976 | 0.851 | 0.909 | 0.842 |
| 1 | 0.897 | 0.972 | 0.795 | 0.875 | 0.800 |
| 2 | 0.866 | 0.909 | 0.862 | 0.885 | 0.726 |
| 3 | 0.928 | 0.977 | 0.875 | 0.923 | 0.86 |
| 4 | 0.845 | 0.872 | 0.773 | 0.819 | 0.689 |
| **Average** | **0.891±0.035** | **0.941±0.048** | **0.831±0.054** | **0.882±0.040** | **0.783±0.074** |

Table 6. Five-Fold cross-validation results on RPI1807 by SAWRPI with XGBoost

| **Fold** | **Acc.** | **Prec.** | **Sen.** | **F1** | **MCC** |
| --- | --- | --- | --- | --- | --- |
| 0 | 0.804 | 0.752 | 0.970 | 0.847 | 0.625 |
| 1 | 0.770 | 0.730 | 0.934 | 0.819 | 0.547 |
| 2 | 0.792 | 0.746 | 0.950 | 0.836 | 0.595 |
| 3 | 0.816 | 0.767 | 0.964 | 0.854 | 0.645 |
| 4 | 0.829 | 0.774 | 0.978 | 0.864 | 0.673 |
| **Average** | **0.802±0.023** | **0.754±0.017** | **0.959±0.017** | **0.844±0.017** | **0.617±0.048** |

## ExtraTrees

Table 7. Five-Fold cross-validation results on RPI369 by SAWRPI with ExtraTrees

| **Fold** | **Acc.** | **Prec.** | **Sen.** | **F1** | **MCC** |
| --- | --- | --- | --- | --- | --- |
| 0 | 0.689 | 0.671 | 0.743 | 0.705 | 0.381 |
| 1 | 0.682 | 0.663 | 0.743 | 0.701 | 0.368 |
| 2 | 0.676 | 0.681 | 0.662 | 0.671 | 0.351 |
| 3 | 0.687 | 0.684 | 0.703 | 0.693 | 0.374 |
| 4 | 0.714 | 0.687 | 0.781 | 0.731 | 0.433 |
| **Average** | **0.690±0.015** | **0.677±0.010** | **0.726±0.045** | **0.700±0.022** | **0.381±0.031** |

Table 8. Five-Fold cross-validation results on RPI488 by SAWRPI with ExtraTrees

| **Fold** | **Acc.** | **Prec.** | **Sen.** | **F1** | **MCC** |
| --- | --- | --- | --- | --- | --- |
| 0 | 0.867 | 0.854 | 0.872 | 0.863 | 0.735 |
| 1 | 0.876 | 0.944 | 0.773 | 0.850 | 0.757 |
| 2 | 0.856 | 0.907 | 0.845 | 0.875 | 0.707 |
| 3 | 0.876 | 0.891 | 0.854 | 0.872 | 0.753 |
| 4 | 0.825 | 0.787 | 0.841 | 0.813 | 0.650 |
| **Average** | **0.860±0.021** | **0.877±0.060** | **0.837±0.038** | **0.855±0.025** | **0.720±0.044** |

Table 9. Five-Fold cross-validation results on RPI1807 by SAWRPI with ExtraTrees

| **Fold** | **Acc.** | **Prec.** | **Sen.** | **F1** | **MCC** |
| --- | --- | --- | --- | --- | --- |
| 0 | 0.957 | 0.951 | 0.972 | 0.962 | 0.913 |
| 1 | 0.969 | 0.97 | 0.975 | 0.972 | 0.938 |
| 2 | 0.961 | 0.947 | 0.986 | 0.966 | 0.923 |
| 3 | 0.972 | 0.967 | 0.983 | 0.975 | 0.944 |
| 4 | 0.968 | 0.967 | 0.975 | 0.971 | 0.934 |
| **Average** | **0.965±0.006** | **0.960±0.011** | **0.978±0.005** | **0.969±0.005** | **0.930±0.012** |

## RandomForest

Table 10. Five-Fold cross-validation results on RPI369 by SAWRPI with RandomForest

| **Fold** | **Acc.** | **Prec.** | **Sen.** | **F1** | **MCC** |
| --- | --- | --- | --- | --- | --- |
| 0 | 0.703 | 0.703 | 0.703 | 0.703 | 0.405 |
| 1 | 0.649 | 0.657 | 0.622 | 0.639 | 0.298 |
| 2 | 0.662 | 0.667 | 0.649 | 0.658 | 0.324 |
| 3 | 0.701 | 0.692 | 0.730 | 0.711 | 0.402 |
| 4 | 0.714 | 0.707 | 0.726 | 0.716 | 0.429 |
| **Average** | **0.686±0.028** | **0.685±0.022** | **0.686±0.048** | **0.685±0.035** | **0.372±0.057** |

Table 11. Five-Fold cross-validation results on RPI488 by SAWRPI with RandomForest

| **Fold** | **Acc.** | **Prec.** | **Sen.** | **F1** | **MCC** |
| --- | --- | --- | --- | --- | --- |
| 0 | 0.929 | 0.955 | 0.894 | 0.923 | 0.858 |
| 1 | 0.887 | 0.946 | 0.795 | 0.864 | 0.777 |
| 2 | 0.866 | 0.925 | 0.845 | 0.883 | 0.731 |
| 3 | 0.918 | 0.976 | 0.854 | 0.911 | 0.841 |
| 4 | 0.856 | 0.875 | 0.795 | 0.833 | 0.709 |
| **Average** | **0.891±0.032** | **0.935±0.038** | **0.837±0.042** | **0.883±0.036** | **0.783±0.066** |

Table 12. Five-Fold cross-validation results on RPI1807 by SAWRPI with RandomForest

| **Fold** | **Acc.** | **Prec.** | **Sen.** | **F1** | **MCC** |
| --- | --- | --- | --- | --- | --- |
| 0 | 0.958 | 0.961 | 0.964 | 0.963 | 0.916 |
| 1 | 0.968 | 0.975 | 0.967 | 0.971 | 0.935 |
| 2 | 0.958 | 0.956 | 0.970 | 0.963 | 0.916 |
| 3 | 0.963 | 0.964 | 0.970 | 0.967 | 0.925 |
| 4 | 0.977 | 0.975 | 0.983 | 0.979 | 0.953 |
| **Average** | **0.965±0.008** | **0.966±0.009** | **0.971±0.007** | **0.969±0.007** | **0.929±0.016** |

## Stacked Ensemble

Table 13. Five-Fold cross-validation results on RPI369 by SAWRPI with Stacked Ensemble

| **Fold** | **Acc.** | **Prec.** | **Sen.** | **F1** | **MCC** |
| --- | --- | --- | --- | --- | --- |
| 0 | 0.743 | 0.720 | 0.797 | 0.756 | 0.489 |
| 1 | 0.669 | 0.651 | 0.730 | 0.688 | 0.340 |
| 2 | 0.703 | 0.692 | 0.730 | 0.711 | 0.406 |
| 3 | 0.707 | 0.701 | 0.730 | 0.715 | 0.415 |
| 4 | 0.714 | 0.687 | 0.781 | 0.731 | 0.433 |
| **Average** | **0.707±0.027** | **0.690±0.025** | **0.753±0.033** | **0.720±0.25** | **0.417±0.054** |

Table 14. Five-Fold cross-validation results on RPI488 by SAWRPI with Stacked Ensemble

| **Fold** | **Acc.** | **Prec.** | **Sen.** | **F1** | **MCC** |
| --- | --- | --- | --- | --- | --- |
| 0 | 0.918 | 0.976 | 0.851 | 0.909 | 0.842 |
| 1 | 0.897 | 0.972 | 0.795 | 0.875 | 0.800 |
| 2 | 0.876 | 0.911 | 0.879 | 0.895 | 0.746 |
| 3 | 0.907 | 0.933 | 0.875 | 0.903 | 0.816 |
| 4 | 0.866 | 0.878 | 0.818 | 0.847 | 0.729 |
| **Average** | **0.893±0.022** | **0.934±0.042** | **0.844±0.037** | **0.886±0.025** | **0.787±0.048** |

Table 15. Five-Fold cross-validation results on RPI1807 by SAWRPI with Stacked Ensemble

| **Fold** | **Acc.** | **Prec.** | **Sen.** | **F1** | **MCC** |
| --- | --- | --- | --- | --- | --- |
| 0 | 0.955 | 0.954 | 0.967 | 0.960 | 0.909 |
| 1 | 0.971 | 0.970 | 0.978 | 0.974 | 0.941 |
| 2 | 0.960 | 0.956 | 0.972 | 0.964 | 0.919 |
| 3 | 0.965 | 0.964 | 0.972 | 0.968 | 0.928 |
| 4 | 0.974 | 0.967 | 0.986 | 0.977 | 0.947 |
| **Average** | **0.965±0.008** | **0.962±0.007** | **0.975±0.007** | **0.969±0.007** | **0.929±0.016** |

# Results of five-fold cross-validation on three datasets with each classifier through other different method of features extracting


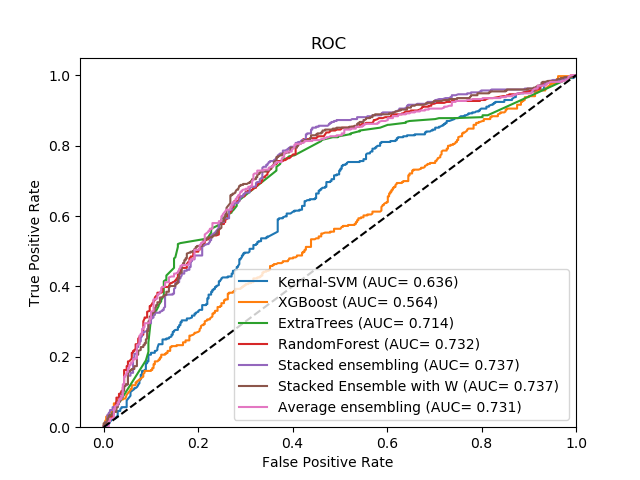


**Supplementary Figure 4.** ROC curves figures of comparing all classifying strategies in RPI369 datasets with Auto-covariance extracting features


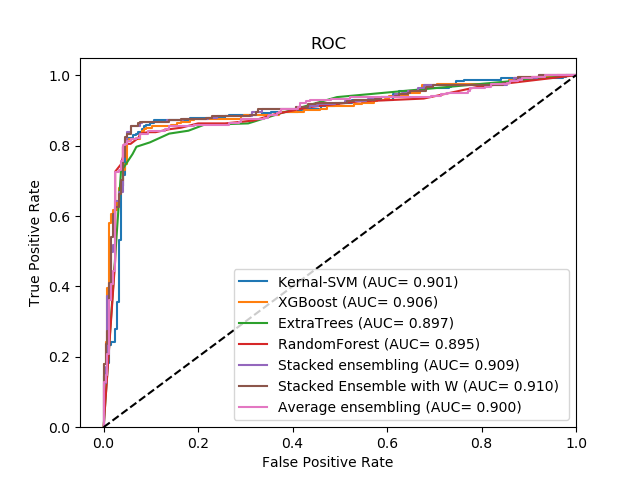


**Supplementary Figure 5.** ROC curves figures of comparing all classifying strategies in RPI488 datasets with Auto-covariance extracting features


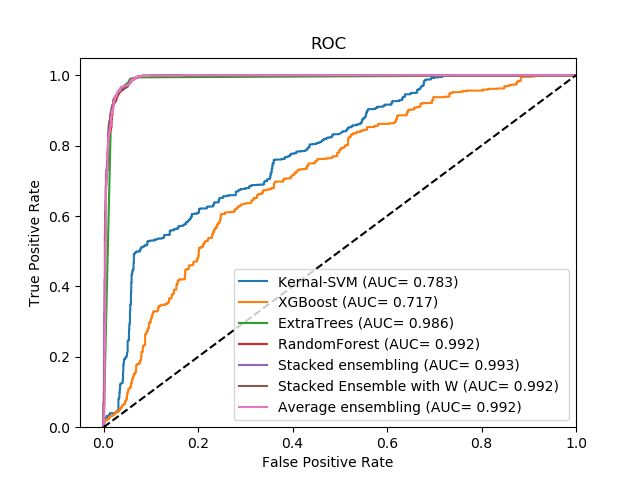


**Supplementary Figure 6.** ROC curves figures of comparing all classifying strategies in RPI1807 datasets with Auto-covariance extracting features


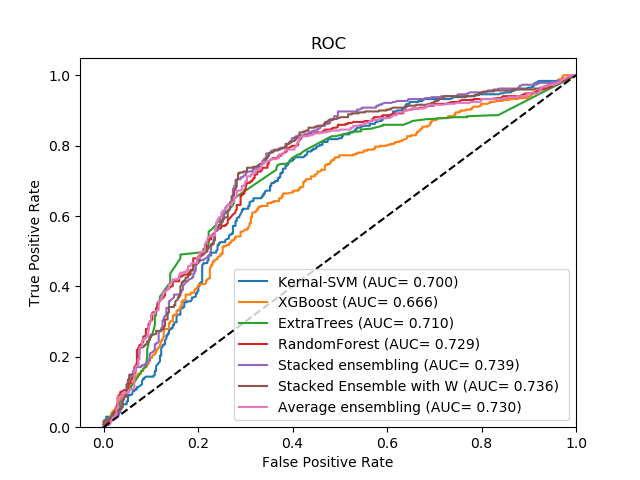


**Supplementary Figure 7.** ROC curves figures of comparing all classifying strategies in RPI369 datasets with Discrete Wavelet transform extracting features


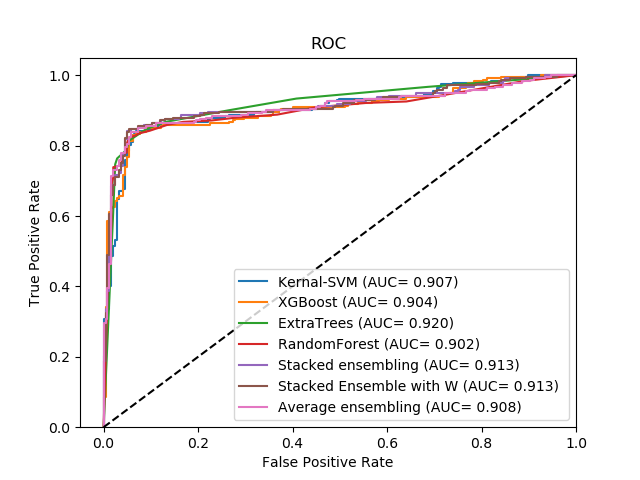


**Supplementary Figure 8.** ROC curves figures of comparing all classifying strategies in RPI488 datasets with Discrete Wavelet transform extracting features


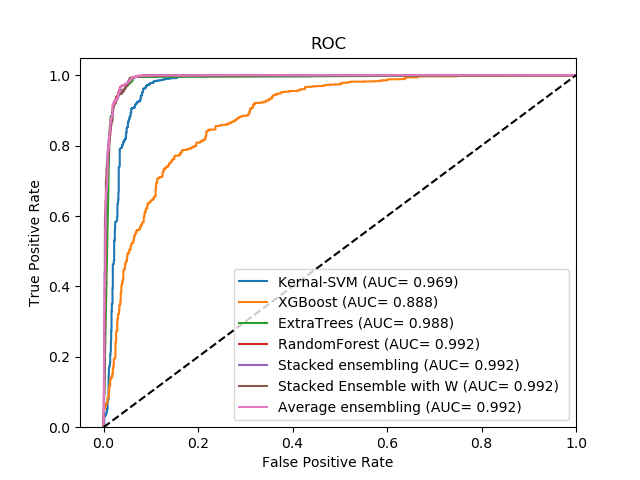


**Supplementary Figure 9.** ROC curves figures of comparing all classifying strategies in RPI1807 datasets with Discrete Wavelet transform extracting features

Table 16. Five-Fold cross-validation results on RPI369 by SAWRPI with Auto-covariance

| **Classier** | **Acc.** | **Prec.** | **Sen.** | **F1** | **MCC** |
| --- | --- | --- | --- | --- | --- |
| SVM | 0.543 | 0.549 | 0.526 | 0.535 | 0.087 |
| XGB | 0.594 | 0.605 | 0.556 | 0.577 | 0.189 |
| ExtraTrees | 0.680 | 0.689 | 0.656 | 0.672 | 0.361 |
| RandomForest | 0.682 | 0.666 | 0.729 | 0.696 | 0.366 |
| Stacked ensembling | 0.701 | 0.687 | 0.737 | 0.711 | 0.402 |
| Stacked ensembling with X | 0.690 | 0.675 | 0.732 | 0.702 | 0.381 |

Table 17. Five-Fold cross-validation results on RPI488 by SAWRPI with Auto-covariance

| **Classier** | **Acc.** | **Prec.** | **Sen.** | **F1** | **MCC** |
| --- | --- | --- | --- | --- | --- |
| SVM | 0.874 | 0.913 | 0.823 | 0.865 | 0.749 |
| XGB | 0.883 | 0.899 | 0.857 | 0.877 | 0.763 |
| ExtraTrees | 0.879 | 0.914 | 0.832 | 0.870 | 0.758 |
| RandomForest | 0.831 | 0.821 | 0.842 | 0.830 | 0.663 |
| Stacked ensembling | 0.893 | 0.923 | 0.852 | 0.886 | 0.786 |
| Stacked ensembling with X | 0.893 | 0.923 | 0.852 | 0.886 | 0.786 |

Table 18. Five-Fold cross-validation results on RPI1807 by SAWRPI with Auto-covariance

| **Classier** | **Acc.** | **Prec.** | **Sen.** | **F1** | **MCC** |
| --- | --- | --- | --- | --- | --- |
| SVM | 0.647 | 0.621 | 0.937 | 0.747 | 0.299 |
| XGB | 0.686 | 0.676 | 0.838 | 0.748 | 0.358 |
| ExtraTrees | 0.961 | 0.965 | 0.965 | 0.965 | 0.921 |
| RandomForest | 0.965 | 0.962 | 0.976 | 0.969 | 0.930 |
| Stacked ensembling | 0.960 | 0.962 | 0.966 | 0.964 | 0.919 |
| Stacked ensembling with X | 0.961 | 0.960 | 0.971 | 0.965 | 0.921 |

Table 19. Five-Fold cross-validation results on RPI369 by SAWRPI with Discrete Wavelet transform

| **Classier** | **Acc.** | **Prec.** | **Sen.** | **F1** | **MCC** |
| --- | --- | --- | --- | --- | --- |
| SVM | 0.627 | 0.654 | 0.545 | 0.593 | 0.260 |
| XGB | 0.660 | 0.677 | 0.613 | 0.642 | 0.323 |
| ExtraTrees | 0.690 | 0.692 | 0.680 | 0.686 | 0.380 |
| RandomForest | 0.683 | 0.667 | 0.732 | 0.697 | 0.368 |
| Stacked ensembling | 0.707 | 0.692 | 0.748 | 0.719 | 0.416 |
| Stacked ensembling with X | 0.706 | 0.689 | 0.751 | 0.718 | 0.414 |

Table 20. Five-Fold cross-validation results on RPI488 by SAWRPI with Discrete Wavelet transform

| **Classier** | **Acc.** | **Prec.** | **Sen.** | **F1** | **MCC** |
| --- | --- | --- | --- | --- | --- |
| SVM | 0.883 | 0.931 | 0.823 | 0.873 | 0.767 |
| XGB | 0.870 | 0.887 | 0.845 | 0.864 | 0.739 |
| ExtraTrees | 0.881 | 0.923 | 0.829 | 0.873 | 0.763 |
| RandomForest | 0.879 | 0.905 | 0.845 | 0.873 | 0.756 |
| Stacked ensembling | 0.893 | 0.932 | 0.843 | 0.885 | 0.786 |
| Stacked ensembling with X | 0.893 | 0.932 | 0.843 | 0.885 | 0.786 |

Table 21. Five-Fold cross-validation results on RPI1807 by SAWRPI with Discrete Wavelet transform

| **Classier** | **Acc.** | **Prec.** | **Sen.** | **F1** | **MCC** |
| --- | --- | --- | --- | --- | --- |
| SVM | 0.808 | 0.765 | 0.945 | 0.846 | 0.623 |
| XGB | 0.941 | 0.932 | 0.966 | 0.948 | 0.882 |
| ExtraTrees | 0.966 | 0.968 | 0.971 | 0.969 | 0.931 |
| RandomForest | 0.962 | 0.958 | 0.975 | 0.966 | 0.923 |
| Stacked ensembling | 0.964 | 0.961 | 0.974 | 0.968 | 0.926 |
| Stacked ensembling with X | 0.965 | 0.961 | 0.977 | 0.969 | 0.929 |

# Average results on three datasets with different classifiers

Table 22. Five-Fold cross-validation average results on RPI369 by different classifier

| **Classifier** | **Acc.** | **Prec.** | **Sen.** | **F1** | **MCC** |
| --- | --- | --- | --- | --- | --- |
| XGB | 0.553 | 0.551 | 0.596 | 0.571 | 0.107 |
| SVM | 0.638 | 0.661 | 0.569 | 0.610 | 0.280 |
| RF | 0.686 | 0.685 | 0.686 | 0.685 | 0.372 |
| ExtraTree | 0.690 | 0.677 | 0.726 | 0.700 | 0.381 |
| Stacked ensembling | 0.707 | 0.690 | 0.753 | 0.720 | 0.417 |
| Stacked ensembling with X | **0.710** | **0.692** | **0.756** | **0.723** | **0.422** |

The bold values represent the higher values each column.

Table 23. Five-Fold cross-validation average results on RPI488 by different classifier

| **Classifier** | **Acc.** | **Prec.** | **Sen.** | **F1** | **MCC** |
| --- | --- | --- | --- | --- | --- |
| XGB | 0.891 | 0.941 | 0.831 | 0.882 | 0.783 |
| SVM | 0.887 | 0.916 | **0.848** | 0.880 | 0.773 |
| RF | 0.891 | 0.935 | 0.837 | 0.883 | 0.783 |
| ExtraTree | 0.860 | 0.877 | 0.837 | 0.855 | 0.720 |
| Stacked ensembling | 0.893 | 0.934 | 0.844 | 0.886 | 0.787 |
| Stacked ensembling with X | **0.895** | **0.938** | 0.844 | **0.888** | **0.791** |

The bold values represent the higher values each column.

Table 24. Five-Fold cross-validation average results on RPI1807 by different classifier

| **Classifier** | **Acc.** | **Prec.** | **Sen.** | **F1** | **MCC** |
| --- | --- | --- | --- | --- | --- |
| XGB | 0.802 | 0.754 | 0.959 | 0.844 | 0.617 |
| SVM | 0.899 | 0.876 | 0.952 | 0.913 | 0.796 |
| RF | 0.965 | **0.966** | 0.971 | 0.969 | 0.929 |
| ExtraTree | 0.965 | 0.960 | 0.978 | 0.969 | 0.930 |
| Stacked ensembling | 0.965 | 0.962 | 0.975 | 0.969 | 0.928 |
| Stacked ensembling with X | **0.967** | 0.961 | **0.981** | **0.971** | **0.934** |

The bold values represent the higher values each column.

# Statistical learning methods verifying the experimental results on RPI1807


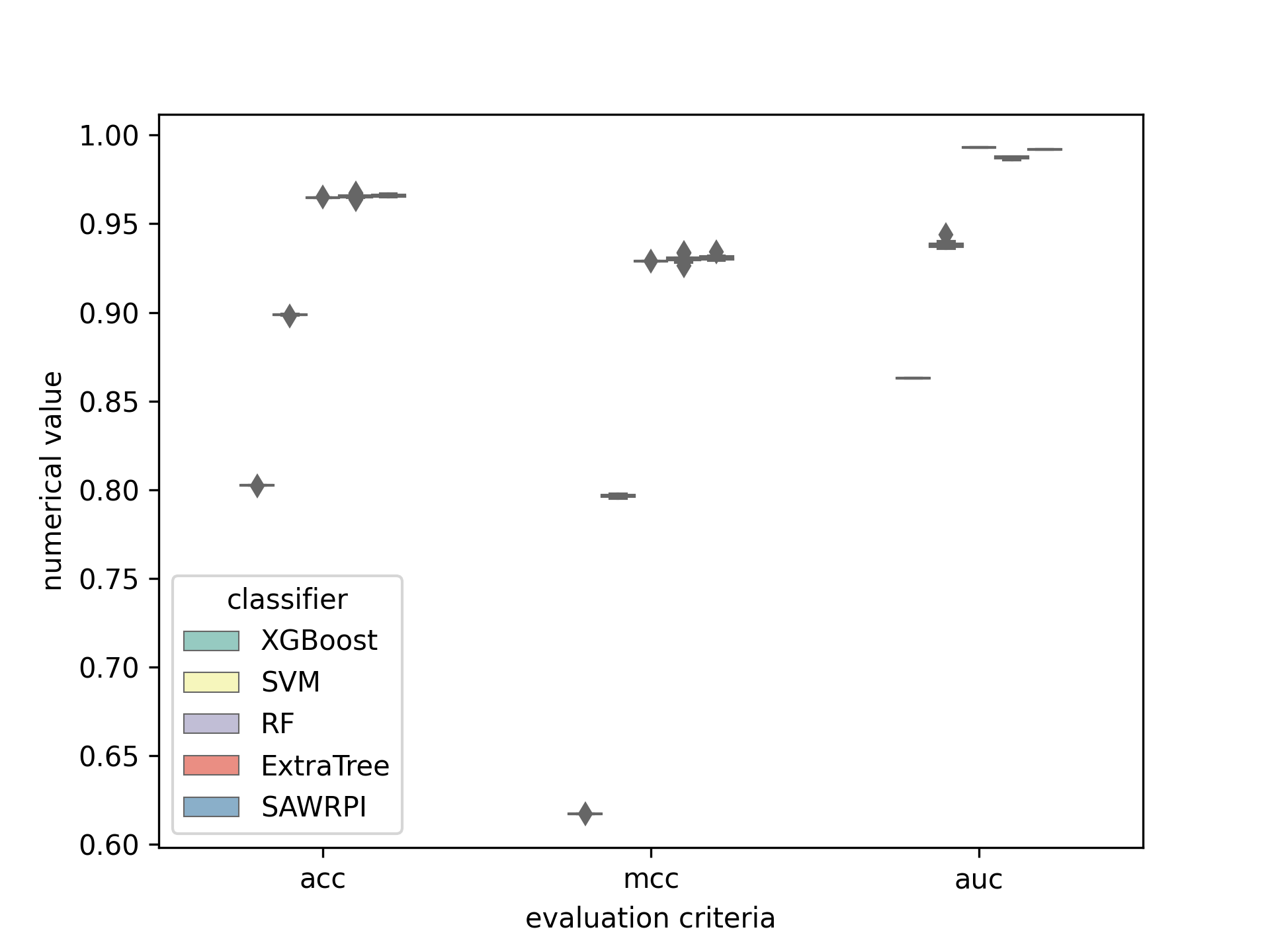


**Supplementary Figure 10.** Experimental results of SAWRPI on RPI1807 datasets with different classifiers.


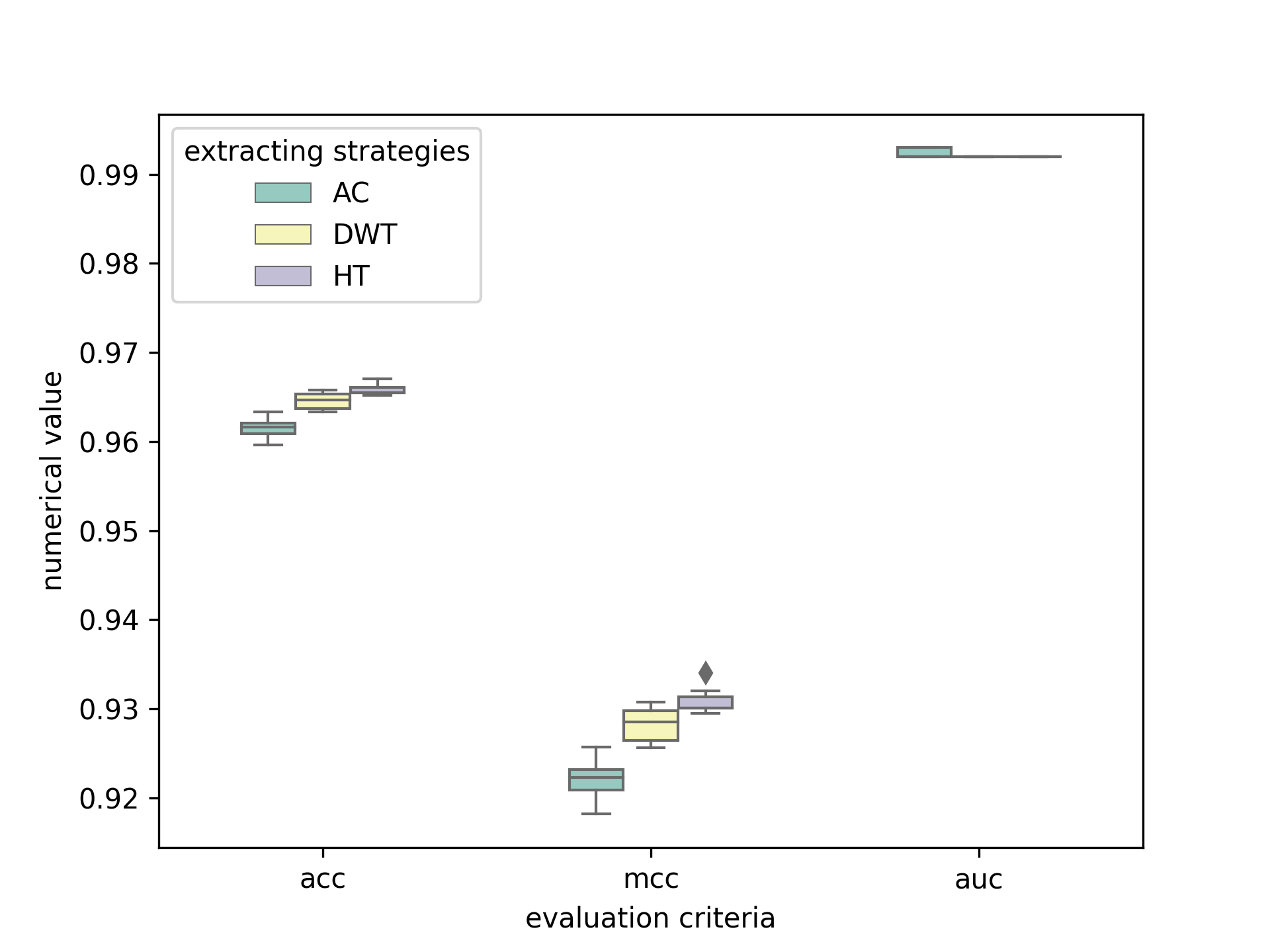


**Supplementary Figure 11.** Experimental results of SAWRPI on RPI1807 datasets with different extracting features strategies.
